# Examining the relationship between local extinction risk and position in range

**DOI:** 10.1111/cobi.12979

**Published:** 2017-11-08

**Authors:** Elizabeth H. Boakes, Nicholas J.B. Isaac, Richard A. Fuller, Georgina M. Mace, Philip J.K. McGowan

**Affiliations:** ^1^ Centre for Biodiversity and Environment Research University College London Gower Street London WC1E 6BT U.K.; ^2^ NERC Centre for Ecology and Hydrology Wallingford Oxfordshire OX10 8BB U.K.; ^3^ School of Biological Sciences University of Queensland Brisbane QLD 4072 Australia; ^4^ School of Biology Newcastle University Newcastle upon Tyne NE1 7RU U.K.

**Keywords:** biodiversity monitoring, dynamic occupancy model, Galliformes, geographic range, land‐use change, multispecies model, spatial ecology, species distribution, cambio en el uso de suelo, distribución de especies, ecología espacial, extensión geográfica, Galliformes, modelo de ocupación dinámica, monitoreo de la biodiversidad

## Abstract

Over half of globally threatened animal species have experienced rapid geographic range loss. Identifying the parts of species’ distributions most vulnerable to local extinction would benefit conservation planning. However, previous studies give little consensus on whether ranges decline to the core or edge. We built on previous work by using empirical data to examine the position of recent local extinctions within species’ geographic ranges, address range position as a continuum, and explore the influence of environmental factors. We aggregated point‐locality data for 125 Galliform species from across the Palearctic and Indo‐Malaya into equal‐area half‐degree grid cells and used a multispecies dynamic Bayesian occupancy model to estimate rates of local extinctions. Our model provides a novel approach to identify loss of populations from within species ranges. We investigated the relationship between extinction rates and distance from range edge by examining whether patterns were consistent across biogeographic realm and different categories of land use. In the Palearctic, local extinctions occurred closer to the range edge than range core in both unconverted and human‐dominated landscapes. In Indo‐Malaya, no pattern was found for unconverted landscapes, but in human‐dominated landscapes extinctions tended to occur closer to the core than the edge. Our results suggest that local and regional factors override general spatial patterns of recent local extinction within species’ ranges and highlight the difficulty of predicting the parts of a species’ distribution most vulnerable to threat.

## Abstract 2

*Article impact statement*: Local and regional factors overwhelm a general tendency for local extinctions to occur at species’ range cores or edges.

## Abstract (es)

Examinación de la Relación entre el Riesgo de Extinción Local y la Posición dentro del Rango de Distribución

**Resumen**

Más de la mitad de las especies amenazadas mundialmente han experimentado una pérdida rápida de su extensión geográfica. La identificación de las partes de la distribución de las especies más vulnerables a la extinción local beneficiaría a la planeación de la conservación. Sin embargo, los estudios prestigiosos dan poco consenso sobre si las extensiones declinan hacia el núcleo o hacia los bordes. Partimos de trabajos previos usando datos empíricos para examinar la posición de las extinciones locales recientes dentro de las extensiones geográficas de las especies, tratar a la posición de la extensión como un continuo y explorar la influencia de los factores ambientales. Añadimos datos de puntos de localidad de 125 especies de galliformes de las regiones Paleártica e Indo‐Malaya a celdas de una cuadrícula de medio grado y de área equitativa y utilizamos un modelo multiespecie de ocupación bayesiana dinámica para estimar la tasa de extinciones locales. Nuestro modelo proporciona una estrategia novedosa para identificar la pérdida de las poblaciones desde dentro de la extensión de las especies. Investigamos la relación entre las tasas de extinción y la distancia a partir del borde de la extensión comprobando si los patrones eran consistentes a lo largo del reino biogeográfico y en diferentes categorías de uso de suelo. En la Paleártica, las extinciones locales ocurrieron más cerca del borde de la extensión que del núcleo tanto en los paisajes sin conversión como en aquellos dominados por el humano. En la Indo‐Malaya, no se encontró un patrón para los paisajes sin conversión, pero en los paisajes dominados por el humano las extinciones tendieron a suceder más cerca del núcleo que del borde de la extensión. Nuestros resultados sugieren que los factores locales y regionales invalidan los patrones espaciales generales de las extinciones locales recientes dentro de la extensión de las especies y resaltan la dificultad de predecir las partes de la distribución de las especies que son más vulnerables a la amenaza.

## Introduction

Substantial geographic range loss has contributed to the extinction vulnerability of over half the approximately 12,000 species of globally threatened animals (IUCN 2016). An ability to forecast which parts of species’ geographic ranges (hereafter range) are most at risk of local extinction would improve predictions of biodiversity loss. The general spatial pattern of range change, specifically whether ranges decline toward the core or edge, has been the subject of much theoretical discussion and empirical research (e.g., Hanski 1982; Channell & Lomolino 2000a; Sagarin et al. 2006; Pironon et al. 2015), but little consensus has emerged. Some studies conclude that species decline toward their range core (Nathan et al. 1996; Donald & Greenwood 2001; Yackulic et al. 2011) and others toward their periphery (Channell & Lomolino 2000a; Farnsworth & Ogurcak 2006; Fisher 2011).

Many studies have focused on understanding the evolutionary mechanisms behind range change, for example by determining the distribution of abundance (e.g., Blackburn et al. 1999; Sagarin & Gaines 2002) or genetic diversity (e.g., Hampe & Petit 2005; Eckert et al. 2008) across species’ ranges. However, the recent scale of anthropogenic processes driving biodiversity loss may now overwhelm these natural patterns (Yackulic et al. 2011; Lucas et al. 2016). Drivers of change such as coastal settlement or deforestation that move contagiously across the landscape could lead to increased vulnerability of range edges (Boakes et al. 2010a). Alternatively, because species’ ranges and ecosystem boundaries are often not identical and the spatial reach of anthropogenic disturbance is larger than ever before (Sanderson et al. 2002), species may be equally vulnerable across their entire range.

Understanding the spatial patterns of range change raises a practical challenge; knowledge of species’ distributions is much less comprehensive than range maps suggest. Studies of geographic range change are restricted and biased by data availability (Boakes et al. 2010b); thus, information on changes in range extent is uneven. Previous studies of decline to core versus periphery (e.g., Channell & Lomolino 2000a, 2000b) have used generalized distribution maps to analyze historic range loss. Although these studies provide insights into past range decline, the coarse spatial resolution of the historic ranges and often dichotomous measure of position in range (i.e., edge or core) limit their statistical power and conclusions that can be drawn. For example, because many species occur in only a small fraction of their extent of occurrence (EOO) (Gaston & Fuller 2009), assessments of range loss based on EOO polygons may identify range loss in places where the species never occurred or overlook significant fragmentation within occupied areas. It would thus be preferable to examine patterns of range loss at a finer spatial resolution while addressing range position as a continuum (Yackulic et al. 2011). Historical species occurrence data are widely scattered in museums, published literature, and unpublished reports, but intensive and directed data gathering can provide comprehensive information on species occurrences over time and space (e.g. Boakes et al. 2010b; Turvey et al. 2015).

We used multispecies dynamic occupancy modeling to analyze a compilation of point‐locality data on the presence of galliforms over the last 2 centuries (Boakes et al. 2010b). We explored links between position in a range and local‐extinction risk for the 125 European and Asian species of Galliformes. Specifically, we tested the hypothesis that local extinction rates are affected by distance to range edge and investigated whether effects are consistent among the Palaearctic and Indo‐Malayan biogeographic realms, human‐dominated versus unconverted land‐use types, and species.

## Methods

### Distributional Data

The data included 125 species of Galliformes (pheasants, quails, grouse, etc.) in the Palearctic and Indo‐Malay biogeographic realms (Supporting Information). Historical distribution data for the Galliformes are of relatively high quality owing to their long association with humans through hunting and religious symbolism (McGowan & Garson 2002) and their attraction for collectors and ornithologists. Almost all species are resident, making range delimitation tractable. Over 25% of Galliformes are threatened (IUCN 2016) and many local extinctions have been reported (BirdLife International 2015).

**Figure 1:**
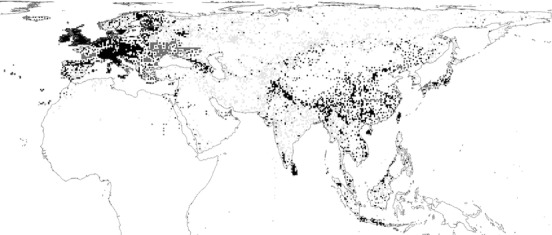
The distribution of the 8551 cells where Galliformes were recorded at least once (black, occurrence recorded before and after 1980, 2512 cells; light grey, recorded only before 1980, 3511 cells; dark grey, recorded only after 1980, 2528 cells).

Point‐locality data, at a resolution of ≤30 minutes (about 50 km) were collected from museums, journal articles, personal reports and letters, banding records, ornithological atlases, and birdwatching trip‐report websites (see Boakes et al. [2010b] for detailed description of sources). Records were included if they could be dated to within 10 years or were known to be pre‐ or post‐1980. Records from non‐native parts of species’ ranges (determined from range maps in McGowan [1994]) were not included other than as a measure of survey effort (see Boakes et al. [2010b] and McGowan et al. [1999] for details). The final database contained 158,714 locality records dating from 1727 to 2008; the median year was 1981 (Supporting Information). Although the data set was compiled as comprehensively as possible, record coverage was unavoidably uneven; the last three decades showed a strong bias toward threatened species and protected areas (Boakes et al. 2010b) and countries rated as relatively “peaceful” (Institute for Economics and Peace 2015; Boakes et al. 2016). We used a grid with cells measuring 48.24 × 48.24 km (i.e., 30‐minute resolution) to aggregate the point‐locality data into a Behrmann equal‐area projection. Grid‐cell size was chosen to maximize spatial resolution within the constraints of the spatial coverage of our data. The data set contained 8551 cells with at least one native species observation (Fig. 1).

### Measuring Distance from Range Edge

No single measure can encapsulate all aspects of geographic range change (e.g., contraction from one edge, fragmentation, collapse to the core). Even defining the edges and core of a range is difficult. Exact range margins do not exist in the sense of a strict border (Gaston 1994). If margins are drawn to exclude outliers, their delineation will be arbitrary, but ranges encompassing all occurrences are disproportionately affected by a few outliers (Quinn et al. 1996). Distance to range edge can be estimated in ovoid ranges by measuring distance from the centroid. However, irregularly shaped ranges present conceptual and practical problems: the centroid can fall close to, or even outside, the inferred border (Sagarin et al. 2006) (Supporting Information). We therefore employed a continuous measure of distance to range edge that was not based on the range centroid or the range border.

To measure distance from range edge, we calculated *D*_0_, the geometric mean distance from the centroid of a cell to the centroids of all other cells containing that species (excluding introductions) (following Blackburn et al. 1999). We derived a species’ historical distribution by aggregating the grid cells in our database with the range polygon from Orme et al. (2005) (Supporting Information). Sometimes occurrence records that we were confident were within the native range fell close to but outside these range polygons. This is to be expected because range maps are approximations and the Orme et al. (2005) polygons are compiled from relatively recent sources.

The *D*_0_ values were standardized to allow comparison across species (Fortin et al. 2005). For each species, *D*_0_ values were divided by the largest value of *D*_0_ for that species to yield the standardized *D*′. A *D*′ value close to 1 indicates a cell is near the range edge, although, depending on range shape, some edges are farther from the center (i.e., edgier) than others. For extremely small‐ranged species, the gridded data meant that all cells were almost equally edgy and thus all have values of *D*′ close to 1 (Supporting Information). These ranges are thus effectively treated as having no core—the center of a small range is closer to the range edge than is the center of a large range. An alternative would be to force the distance to edge measure to run from 0 to 1, but this vastly overinflates the variance of the distance‐to‐edge measure for small‐ranged species.

### Biogeographic Realm and Landscape Type

The Palearctic biogeographic realm has a longer history of anthropogenic disturbance than Indo‐Malaya (Ellis et al. 2010); thus, we hypothesized that the 2 realms might exhibit different spatial patterns of range loss. Using the cells’ centroids, we assigned cells to a realm as defined by Olson et al. (2001).

We investigated whether ranges follow the same pattern of decline in unconverted and human‐dominated landscapes. We classified cells as unconverted or human‐dominated based on a threshold of one‐third of land having been converted for human use before 1970. We used the HYDE 2.0 Land Use Data (Klein Goldewijk 2001) because it gave the closest approximation of anthropogenic disturbance available for 1980 (Supporting Information). It has been suggested that increasingly rapid loss of biodiversity occurs following landscape conversion of over 30% (Andrén 1994), and this threshold has the additional advantage of a similar number of cells in the 2 sets (1319 human‐dominated cells; 1199 unconverted cells). The uncertainty associated with historical modeled land‐use data (Klein Goldewijk & Verburg 2013) is small for recent decades.

### Multispecies Occupancy Model

Estimating extinction is a major challenge because an absence of sightings does not necessarily indicate extinction, especially if search effort or detectability is low (Boakes et al. 2015). Most of our data were opportunistically collected presence‐only data (as opposed to systematic presence–absence survey data), and there are likely to be many pseudoabsences—cases where a species was present but not recorded—as well as other spatial and temporal biases (Isaac & Pocock 2015). Hierarchical Bayesian occupancy‐detection (BOD) models are robust to these biases if they contain parameters to describe the data‐collection process (van Strien et al. 2013; Isaac et al. 2014). In these models, the occupancy of each grid cell (presence or absence) is separated statistically from the data‐collection process (detection vs. nondetection); specifically, observations are conditional on the species being present.

Our modeling framework required us to define a temporal resolution at which to estimate occupancy within which repeated surveys can be identified. We worked with 2 periods, before and after 1980, the median year of observation. A larger number of periods would have reduced the precision of our occupancy estimates. Having defined this threshold, we excluded grid cells that contained no information about relative extinction rates (3511 cells with no observations after 1980 and 2528 cells lacking data before 1980). Our analysis was based on 2512 cells with observations in both periods. No cells contained records of Arabian Chukar (*Alectoris melanocephala*) or Philby's Rock Partridge (*Alectoris philbyi*) from both periods, so these species were excluded from further analyses. There were 123 remaining species. We used a temporal precision of one year to define repeat surveys within grid cells. Our final data set contained 18,492 surveys, which equated to an average of 3.68 surveys per grid cell per period (SD 4.88).

We employed a multispecies dynamic BOD model similar to Woodcock et al.’s (2016). The model is dynamic (Royle & Dorazio 2008) in that extinction and colonization of individual grid cells is modeled explicitly (Eq. 1) and multispecies (Ruiz‐Gutierrez et al. 2010) in that we fitted a single model to the full data set with species‐specific parameter estimates. The model consisted of 2 submodels: state and detection. The state submodel defined the occupancy (presence or absence) of 123 species on 2,512 grid cells in each of the 2 periods. We included only the 14,256 species‐cell combinations within the range polygon of each species (i.e., the state submodel had 28,512 elements). The detection submodel defined the probability, per survey, of detecting a species that is present. The data were 112,485 binary observations (one per survey‐species combination) on whether the species was detected or not. The model then estimated the most likely distribution of parameters, given both the data and the condition that species can be detected only if present.

The expected value of z*_i,j,_*_2_ (occupancy of species *i* in grid cell *j* in the second period [i.e., after 1980]) was modeled as a function of occupancy in the first period, z*_i,j,_*_1_. Unoccupied cells could be colonized with species‐specific probability *γ_i_*, whereas occupied cells persisted with a probability *φ_i,j_* (extinction rates were inferred as 1 – *φ*). Occupancy in the second period was a Bernoulli trial with an expected value defined by

(1) $$E\left[z_{i,j,2}\right]=z_{i,j,1}\phi_{i,j}+(1-z_{i,j,1})\gamma_{i}.$$

Population persistence, *φ_i,j_*, was modeled as a linear function of position in range, *D*′, and 2 parameters, *α_i,j_* and *β_i,j_*:

(2) logit ϕi,j=αi,j+βi,j(Di,j′−0.5).

We subtracted 0.5 from the *D*′ scores to avoid confounding our estimates of intercept (*α_i,j_*) and slope (*β_i,j_*) effects. Thus, *β_i,j_* is the difference in persistence between the range edge and center; *α_i,j_* is the persistence rate at some notional point in between. Both *α_i,j_* and *β_i,j_* are composites made up of components for realm (*R*), conversion status (*C*), and species:

(3) $$\alpha_{i,j}=\alpha_{0}\left(1-R_{j}\right)+\alpha_{1}R_{j}+\alpha_{2}\left(1-R_{j}\right)C_{j}+\alpha_{3}R_{j}C_{j}+u_{i}$$

(4) $$\beta_{i,j}=\beta_{0}\left(1-R_{j}\right)+\beta_{1}R_{j}+\beta_{2}\left(1-R_{j}\right)C_{j}+\beta_{3}R_{j}C_{j}+v_{i},$$

where *R_i_* takes the value 0 for cells in the Palearctic and 1 in Indo‐Malaya; *C_i_* takes the value 0 for unconverted cells and 1 for human‐dominated cells. Thus, *α* parameters are intercept terms and *β* parameters define slopes; positive slopes indicate higher persistence at the range edge (i.e., extinction is concentrated in the center) and negative values indicate the converse (extinction is edge prone); *u* and *v* are species‐specific random effects (normally distributed with a mean of 0). Table 1 explains these parameters in more detail. Parameters *β*_0_–*β*_3_ are relevant to our hypotheses about the degree to which the edge proneness of extinction varies with respect to biogeographic realm and conversion status. Values of *v_i_* are relevant to our question about the degree to which edge proneness varies among species.

**Table 1:** Definitions of the parameters in the multispecies occupancy model of local extinction rates of Galliformes

Term	Definition
*φ_i,j_*	probability that a population extant before 1980 persists after that date
*γ_i_*	species‐specific probability that an unoccupied grid cell becomes colonized after 1980
*α*_0,_ *α*_1_	mean intercept: log‐odds of persistence for the average species at unconverted cells in Palearctic (*α*_0_) and Indo‐Malaya (*α*_1_) that are midway between the range edge and core (*DD*′ = 0.5)
*β*_0,_ *β*_1_	mean slope: difference between range edge and core in the log‐odds of persistence for unconverted cells in Palearctic (*β*_0_) and Indo‐Malaya (*β*_1_)
*α*_2,_ *β*_2_	difference in intercept (*α*_2_) and slope (*β*_2_) between human‐dominated and unconverted cells in the Palearctic (for the average species)
*α*_3,_ *β*_3_	as *α*_2_ and *β*_2_ for Indo‐Malaya
*δ*_1_ *_i_*	log odds that a single‐species list is a survey of species *i*
*δ*_2_ *_i_*_,_ *δ*_3_ *_i_*	species‐specific changes in log odds of detection with survey effort
*δ*_t_	difference in log odds of detection before and after 1980

Our detection submodel states that the *k*th survey to a cell occupied by species *i* will yield an observation with probability *p_i,k_*. We defined a survey as the set of unique records from a particular cell:year combination. We modeled this probability as a function of the total number of species recorded on that survey because this provides a convenient measure of sampling effort (Szabo et al. 2010). Specifically, *p_i,k_* is a function of 2 binary variables indicating whether the survey produced a short (2 or 3 species) or long (>3 species) species list (van Strien et al. 2013):

(5) logit pi,k=δt+δ1i+δ2i∗ shor tk+δ3i∗ lon gk.

This formulation (Table 1) treats short lists, long lists, and single‐species surveys as separate data sets with different statistical properties (van Strien et al. 2013) and is not based on the assumption that all surveys record complete lists (Isaac & Pocock 2015). Parameters *δ*_1_–*δ*_3_ carry the subscript *i*, indicating that species are allowed to vary in their detection probability as random effects.

We fitted the model described by Eqs. 1, 2, 3, 4, 5 in a Bayesian framework in the BUGS language implemented in JAGS (Plummer 2014) via the R package jagsUI (Kellner 2014). The BUGS code describing the model and results of a model‐validation exercise are in Supporting Information. We used minimally informative priors and ran the model for 25,000 iterations following a burn‐in of 250,000 on three chains with a thinning rate of 10. Gelman–Rubin statistics indicated satisfactory convergence by this point (all Rhat values <1.05 for all parameters and Rhat <<1.01 for the vast majority). We report three types of statistics to describe our model parameters: mean of the posterior distribution, its standard deviation, and proportion of the posterior distribution that has the same sign as the mean. This value (*f*) is our confidence that the parameter is either positive or negative. It always lies in the range 0.5 ≤ *f* ≤ 1: a value of 0.67 indicates that two‐thirds of the posterior lie one side of 0 and one‐third lie on the other side (i.e., the odds of the parameter taking a particular sign are 2:1).

For each grid cell, we extracted a distribution of species richness values before and after 1980 and used the means of these posterior distributions to calculate change in richness. We calculated species richness for each grid cell in each iteration of the model and thus knew the proportion of iterations in which species richness was higher after 1980 than before. This yielded a continuous measure of confidence about changes in species richness.

### Species’ Range Size and Edge Proneness of Local Extinctions

We investigated whether species’ extinction risk and the degree to which extinctions are concentrated at the range edge (edge‐proneness [*v_i_*]) varies with range size. Larger ranges might confer more protection on their core than smaller ranges. In 7 instances, we amended Orme et al.’s distribution maps to match the IUCN (2016) taxonomy.

Species’ average extinction risk (after controlling for biogeographic realm and conversion history) was measured as 1 − *u_i_* (Eq. 3); edge proneness was measured as *v_j_* (Eq. 4), with positive numbers indicating extinctions were concentrated toward the range center (compared with the average species and after controlling for realm and conversion history); and negative numbers indicating extinctions were concentrated toward the range edge.

We did not conduct a formal test of the interrelationship between *u*_j_, *v*_j_, and geographic range size because the test would be subject to multiple forms of nonindependence. In addition to the phylogenetic nonindependence associated with interspecific comparative tests, our estimates of *u_j_* and *v_j_* were derived from a model in which *D*′ is the independent variable, and *D*′ is not independent of range size. Moreover, the main axes of variation in extinction risk (biogeographic realm and conversion history) were removed, so the test statistic would be misleading. Rather, we made a qualitative comparison to identify species with unusual combinations, especially those species with high rates of extinction concentrated in 1 part of the range.

## Results

Actual values of distance to range edge, *D*′, ranged from 0.199 to 1 (mean of 0.564 [SD 0.171], median of 0.537). Values of *D*′ for the different species co‐occurring within a cell varied considerably (Supporting Information).

**Figure 2:**
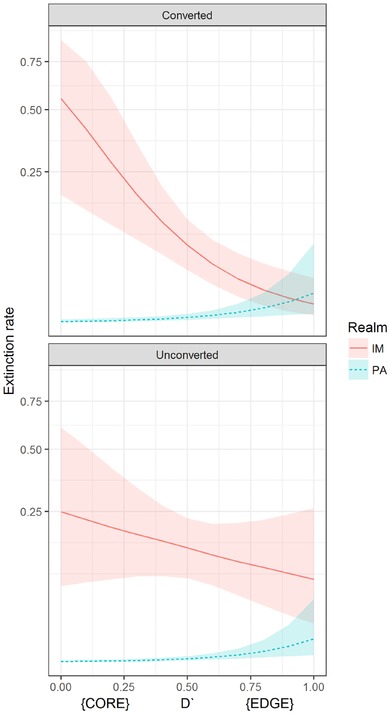
The relationship between extinction rates within cells (calculated as 1‐persistence rates) and their position in range (*D*′) among realms (PA, Palearctic; IM, Indo‐Malaya) and land‐use type (unconverted and human dominated). The data are fitted values from our multispecies dynamic occupancy model and represent the average species’ response (lines, median of the posterior distribution; shading, 80% credible intervals).

**Figure 3:**
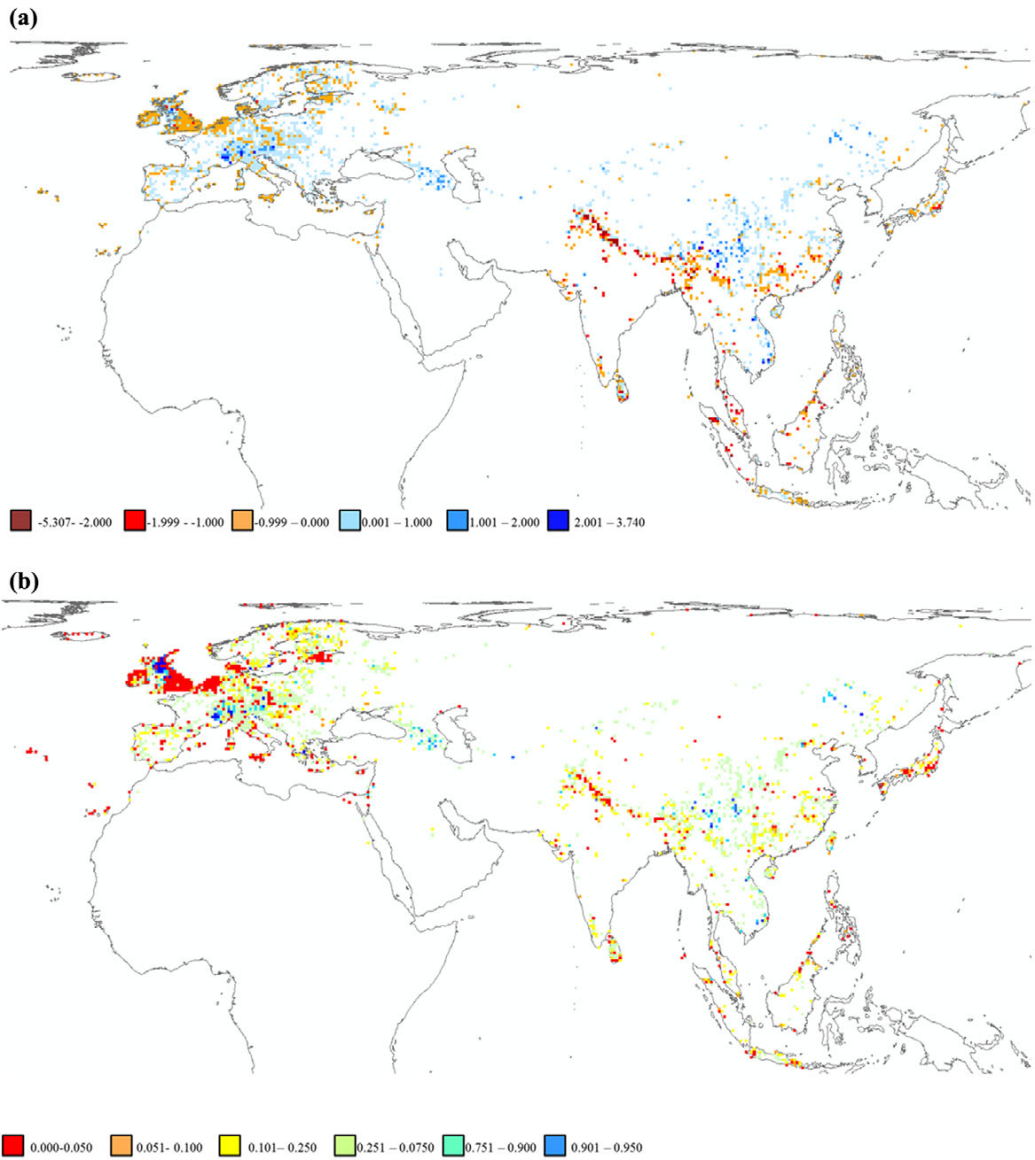
(a) Estimated changes in Galliformes species richness for each grid cell (red tones, loss of species richness; blue tones, gain in species richness). (b) Spatial variation in uncertainty associated with changes in species richness (red tones, values close to zero, high confidence for net loss of species; blue tones, high values, high confidence for net gain of species).

Overall, extinction rates were much lower in the Palearctic than Indo‐Malaya (*α*_0_ >> *α*_1_) (Table 2 & Fig. 2). The effect of landscape conversion on average extinction was small compared with the difference between realms (*α*_2_ << *α*_0_; *α*_3_ << *α*_1_). In unconverted landscapes, extinctions occurred closer to the range edge in the Palearctic (*β*_0_ was negative) (Table 2) but were relatively independent of range position for Indo‐Malayan cells. The value of *β*_1_ was small, and we had low confidence that the effect was different from 0 (Table 2). Human‐dominated landscapes in the Palearctic were edge prone to a similar degree as unconverted landscapes. The value of *β*_2_ was small, and we had low confidence the effect was different from 0 (Table 2). However, human‐dominated landscapes in Indo‐Malaya had higher extinction rates near the center of the range; *β*_3_ was large and positive (Table 2). These findings are plotted in Fig. 2. High relative extinction rates occurred in the Himalayas and the Malay Archipelago (Fig. 3).

**Table 2:** Posterior distribution of parameter values from the multispecies occupancy model modeling local extinction rates of Galliformes

Parameter^a^	Mean	SD	2.5%	97.5%	Rhat^b^	*f*
*α*_0_	9.067	0.997	7.335	11.128	1.048	1
*α*_1_	1.821	0.499	0.943	2.891	1.014	1
*α*_2_	−0.856	0.325	−1.503	−0.224	1.018	0.996
*α*_3_	0.882	0.314	0.291	1.526	1.001	0.998
*β*_0_	−7.719	3.309	−13.919	−1.051	1.004	0.989
*β*_1_	1.429	2.215	−2.776	5.970	1.007	0.737
*β*_2_	0.766	1.292	−1.825	3.242	1.013	0.734
*β*_3_	4.425	2.040	0.500	8.589	1.002	0.984
*Γ*	0.487	0.324	−0.123	0.272	0.477	0.941
*δ*_t_	0.076	0.020	0.038	0.115	1.002	1
*δ*_1_	−1.487	0.084	−1.651	−1.322	1.000	1
*δ*_2_	1.041	0.068	0.908	1.178	1.000	1
*δ*_3_	2.188	0.100	1.994	2.386	1.000	1

a Parameters *α*_0_–*α*_3_ and *β*_0_–*β*_3_ relate to persistence probabilities (Eqs. 2, 3, 4); *γ* relates to the colonization probability (Eq. 1), and *δ* parameters relate to the conditional probability of detection (Eq. 5). Both *γ* and *δ* are reported as means across species.

b Gelman–Rubin convergence statistic, and *f* is the proportion of the posterior distribution that has the same sign as the mean.

**Figure 4:**
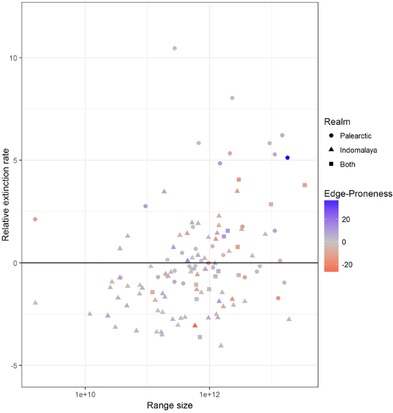
Relative extinction rates (1 − *u_j_*) against species’ geographic range size and tendency toward extinction at the edge of the range (i.e., edge proneness of species’ extinctions [*v_j_*]) (positive values, extinctions concentrated toward the range center). Species‐specific random effects *u_j_* and *v_j_* are estimated as departures from the overall relationships shown in Fig. 2.

There was a weak relationship, at best, between species’ edge proneness and geographic range size (Fig. 4), although species with relatively high extinction rates were all moderately widespread Palearctic species. Extinction rates were low in the Palearctic in absolute terms. However, the relationship identified some key species with interesting dynamics. The bluest point on Fig. 4 represents the Red Grouse (*Lagopus lagopus*), a widespread species that appears to be declining within its range. Its extinction rate was relatively high (for the Palearctic), and it had the overall highest value of *v_j_*, indicating that extinction events were concentrated at the core. By contrast, the reddest point on Fig. 4 represents the median‐sized range of the Necklaced Hill‐partridge (*Arborophila torqueola*). This lowest value of *v_j_* indicated extinctions were concentrated at the range edge. Although this species had one of the lowest relative extinction rates, it is restricted to Indo‐Malaya, where the absolute extinction rates were high (Fig. 2).

## Discussion

Our study of the relationship between spatial properties of species’ ranges and local extinction rates showed that the relationship differed between biogeographic realms and, in Indo‐Malaya, between unconverted and human‐dominated landscapes. Relative extinction rates did not appear to be affected by geographic range size, although species with particularly high relative extinction rates tended to have relatively large geographic ranges.

Our findings raise several important issues for conservation. First, local extinctions in both unconverted and human‐dominated landscapes occurred closer to the edge in the Palearctic and had either no pattern or were farther from the edge in Indo‐Malaya, depending on landscape. The 2 biogeographic realms have different histories of anthropogenic transformation. By 1700 Europe was mostly transformed, but Asia was only beginning its transformation into the intensive cropland and village anthromes that would predominate in the 20th century (Ellis et al. 2010). The intensity of extinction drivers differs between the realms; wildlife extraction in Southeast Asia is estimated to be at 6 times the sustainable rate (Bennett 2002), and current deforestation continues at exceptionally high rates (Sodhi et al. 2004). One explanation of the difference between realms thus might be that local extinctions are mainly central during the early stages of decline and switch to the edge later. Alternatively, the difference might reflect spatial patterns in the drivers of range loss and their severity. The realms’ different biotic compositions might also be a factor (Yackulic et al. 2011). Indo‐Malaya has a more complex geometry; coastal edge often occurs in the center of species’ geographic ranges; *D*′ may have been less representative of range edge in these cases (Supporting Information).

Within Indo‐Malaya, species’ range change differed between land‐use types. In unconverted landscapes, no pattern was seen, but in human‐dominated landscapes local extinctions tended to occur farther from the range edge. This result could be explained by differences in the distribution and intensity of anthropogenic pressures. Hunting is more likely to focus on areas of high species abundance, which are generally scattered across a species’ range (Sagarin & Gaines 2002); thus, one might not expect local extinctions caused by hunting to show a pattern with respect to distance to edge. In contrast, habitat loss might be more likely to cause contraction from an edge inward. More research is needed regarding the placement and intensity of threats within species’ ranges.

The relationship between landscape conversion history, range position, and local extinction rate was more complicated than we anticipated. In the Palearctic, the relationship met our expectation that human‐dominated landscapes have higher extinction rates and that the relationship with position in range would be the same in both types of landscape. By contrast, the pattern in Indo‐Malaya was that overall extinction rates were similar (although slightly lower in human‐dominated landscapes) but concentrated at range centers in human‐dominated landscapes. Our finding of similar extinction rates in both landscapes has implications for models in which land‐cover type (measured or inferred) is used as a proxy for species occurrence or extinction risk (e.g., species‐distribution modeling, protected‐area planning, and IUCN Red List assessments that use habitat loss as a surrogate for population decline).

We found no apparent relationship between relative extinction rate and range size (Fig. 4). However, the Palearctic species undergoing the highest relative extinction rates were all nonthreatened, wide‐ranging species, several of which (e.g. Red Grouse, Black Grouse [*Lyrurus tetrix*], Western Capercaillie [*Tetrao urogallus*], and Barbary Partridge [*Alectoris barbara*]) had high positive values of edge proneness, meaning extinctions tended to occur away from the range edge. Such declines in more central parts of species’ ranges will not be picked up by measures of EOO (Gaston & Fuller 2009); thus, monitoring programs for wide‐ranged species need to be sensitive to central range loss. The three species with the highest relative extinction rates in Indo‐Malaya were the Black Francolin (*Francolinus francolinus*), the Common Quail (*Coturnix coturnix*), and the Rock Bush‐quail (*Perdicula argoondah*). In contrast to the predominate pattern in Indo‐Malaya, these species exhibited high negative values of edge proneness, meaning extinctions tended to occur near the range edge and, along with the Necklaced Hill‐partridge, thus correspond to the classical contraction‐to‐the‐center paradigm. Like their Palearctic counterparts, these species with the highest relative extinction rates are not considered threatened. Such declines must not be overlooked, lest today's common species become tomorrow's threatened species.

In light of our finding that the pattern of range loss is affected by the local factors of biogeographic realm and by land‐use type, the discrepancy between the conclusions of previous studies of range loss with regard to contraction to core or edge (e.g., Channell & Lomolino 2000a; Fisher 2011; vs. Donald & Greenwood 2001; Yackulic et al. 2011), not all of which controlled for these factors, is unsurprising. Should patterns of decline be scale dependent (Thomas et al. 2008), discrepancies between studies would also be expected. Indeed, vulnerability to extinction is almost certainly even more complex than the interactions between position in range, biogeography, and land‐use type that we found. For example, Dos Anjos et al. (2010) found the interaction of endemism and position in range predicts vulnerability, whereas Yackulic et al. (2011) found biome a better predictor of vulnerability than position in range.

Identifying the core and edge of an irregularly shaped range is not simple. We treated the core of small ranges as being close to the range edge. However, there were many range shapes for which a relatively low *D*′ occurred at or near a range edge. One alternative would have been to measure the distance to the nearest border but, as we explained in Methods, defining such a border is in itself problematic and, as *D*′ shows, some edges can be viewed as less edgy than others. How to deal with complex range shapes remains an open question as does the effect of range shape on a species’ vulnerability.

Our analysis was limited by the distribution of our point‐locality data, particularly a lack of recent observations from eastern Europe, northern Asia, and central India (Fig. 1). We were also limited to studying losses of whole populations from grid cells. Changes in occupancy are likely to lag behind changes in abundance (Rodriguez 2002), and we could not model extinctions of species that were observed after 1980 but subsequently became extinct. This lack of baseline data on many species will make understanding of future range or abundance changes extremely difficult. More importance must be placed upon biodiversity data collection and curation. Technological advances coupled with the developmentof statistical methods that can cope with opportunistic and noisy data mean that citizen science is being used in an increasing variety of ways to document species distributions and abundances and apply such data to ecological research (August et al. 2015; Powney & Isaac 2015). We encourage the use of citizen science in addressing issues of spatial and taxonomic bias in biodiversity data globally.

Our approach provides a template for exploring how extinction risk varies in space. We found no overall tendency toward either of the dominant paradigms of range collapse, suggesting that local factors predominate in determining local extinction risk (Cowlishaw et al. 2009). However, we found it is possible to identify widespread species undergoing high rates of population loss within their range core. We anticipate that these insights will be increasingly valuable as the focus of conservation science moves away from rare species in protected areas (Mace 2014).

## Supporting information

Species parameters (Appendix S1), the cumulative number of records over time (Appendix S2), examples of range centroids (Appendix S3), examples of the distributions of point locality data, Orme et al.’s (2005) range polygons and the distributions of *D*′ values (Appendix S4), the HYDE 2.0 land‐use categories (Appendix S5), model validation (Appendix S6), the spatial distribution of *D*′ values (Appendix S7), and the dynamic occupancy model code (Appendix S8) are available online. The authors are solely responsible for the content and functionality of these materials. Queries (other than absence of material) should be directed to the corresponding author.

Supporting Information

Supporting Information
