# Supplementary material for: Examining the relationship between local extinction risk and position in range
Source: Conserv Biol. 2017 Nov 8;32(1):229–39. doi: 10.1111/cobi.12979 (PMC6849610; doi:10.1111/cobi.12979)
Supplement: Supplementary file 1 — Species parameters (Appendix S1), the cumulative number of records over time (Appendix S2), examples of range centroids (Appendix S3), examples of the distributions of point locality data, Orme et al.’s (2005) range polygons and the distributions of D′ values (Appendix S4), the HYDE 2.0 land‐use categories (Appendix S5), model validation (Appendix S6), the spatial distribution of D′ values (Appendix S7), and the dynamic occupancy model code (Appendix S8) are available online. The authors are solely responsible for the content and functionality of these materials. Queries (other than absence of material) should be directed to the corresponding author. [file COBI-32-229-s001.docx]

**TITLE**: Examining the relationship between local extinction risk and position in range

Appendix S2. The cumulative number of records over time.

Appendix S3. An illustration to show how the range centroid has little ecological meaning for non-ovoid ranges. While the centroid of the orange-necked hill-partridge’s (*Arborophila davidi*) roughly ovoid range (a) is far from the range edge, the centroid of the Barbary partridge (*Alectoris barbara*) (b) is on the edge of its largest contiguous block of range and the centroid of the common peacock (*Pavo cristatus*) actually outside its range (c). Range polygons taken from Orme et al. ([2005](#_ENREF_2)), centroids are shown by a black dot, ranges by the shaded in grey area.


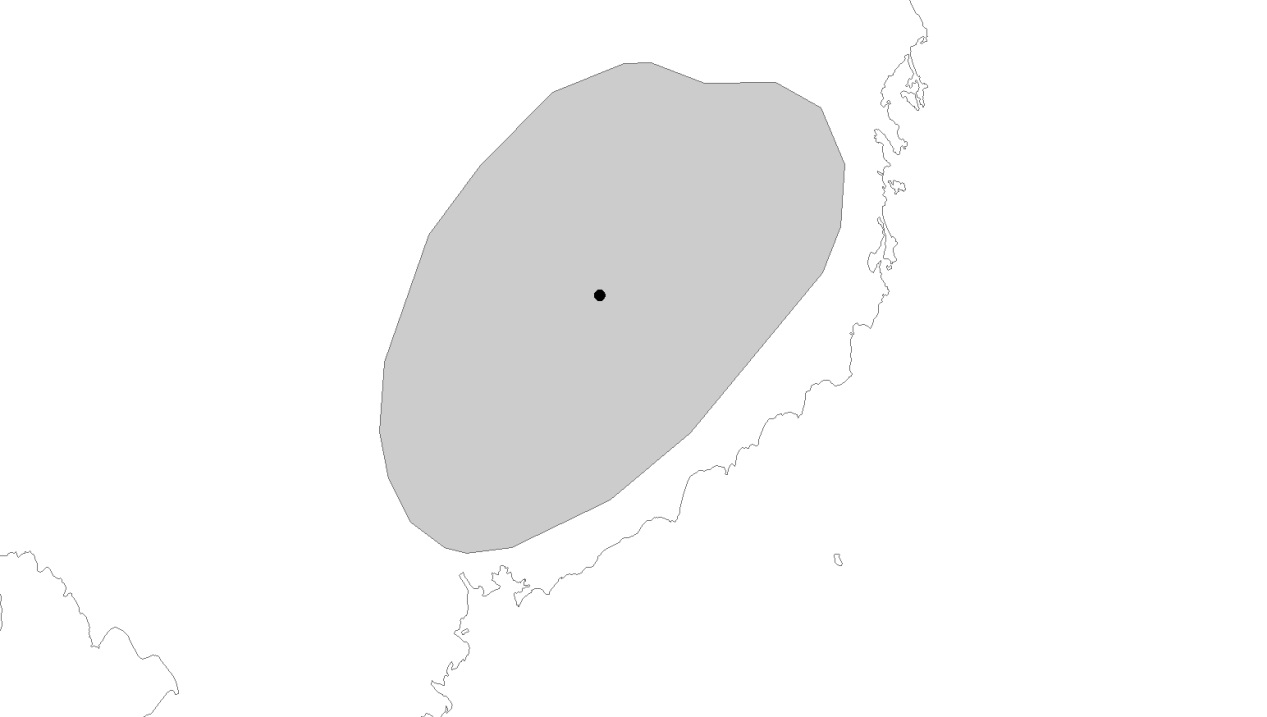


a)


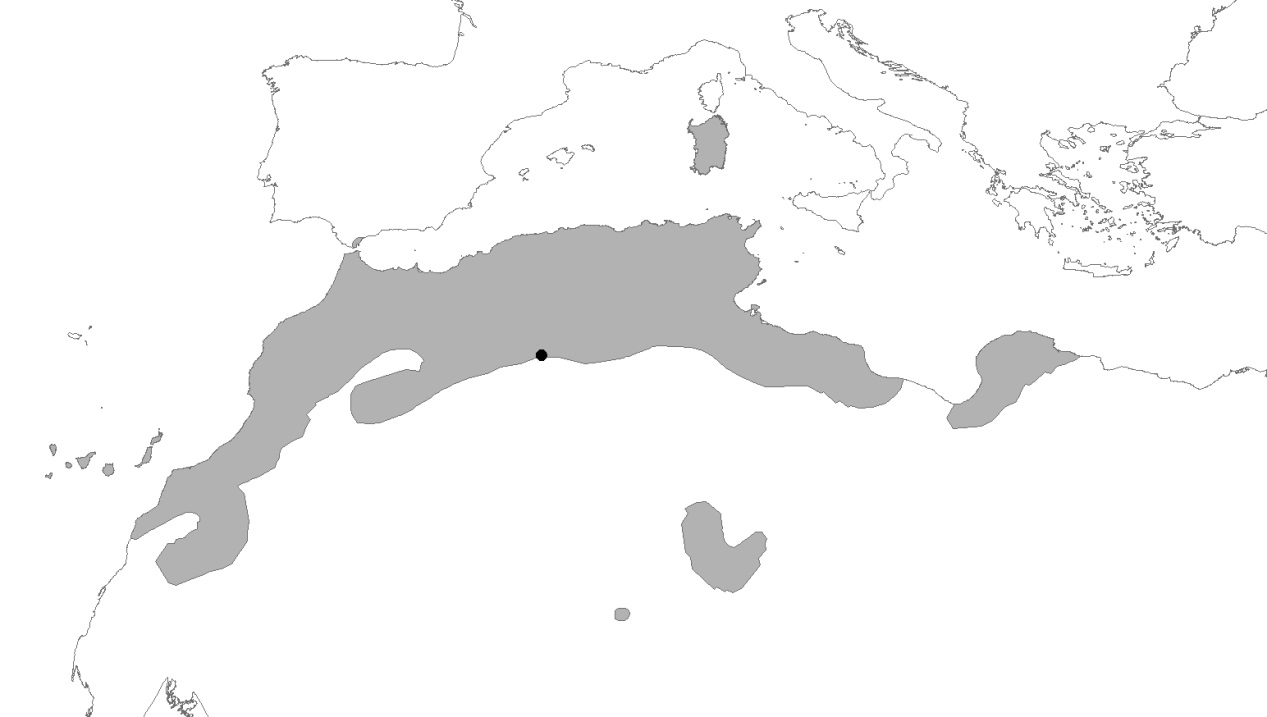


b)


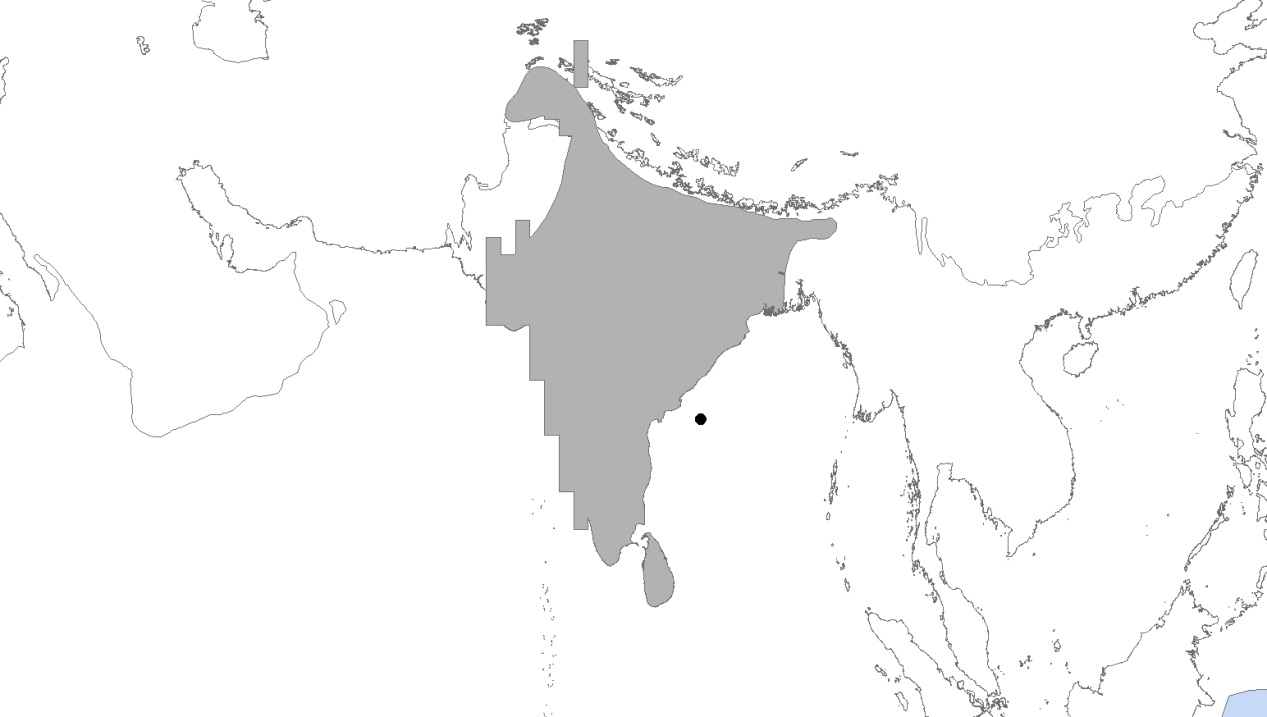


c)

Appendix S4. Aggregations of the gridded point locality data and range polygon data ([from Orme *et al.*, 2005](#_ENREF_2)) showing values of *D^’^* for a) the Western Capercaillie (*Tetrao urogallus*), b) the Common Peafowl (*Pavo muticus*), c) the Crested Wood Partridge (*Rollulus rouloul)* and d) the Himalayan Quail (*Ophrysia superciliosa*). Point locality data (cells containing black dots) can slightly exceed the boundaries of the Orme *et al.* ([2005](#_ENREF_2)) range polygons and, particularly in the case of the capercaillie, do not occur evenly across the ranges. Within each species, the mean distance from each cell in the aggregated data to every other cell was calculated to give *D_0_*. These values were then divided by the largest value of *D_0_* for each species to give a standardised measure *D^`^.* It can be seen that some edges are more ‘edgy’ than others – for example, the edges of the mid-portion of the Capercaillie’s range have lower values of *D`* than the eastern and western edges and thus are seen as ‘core’. The Crested Wood Partridge’s ‘core’ range occurs along coastal edge. All cells in the Himalayan Quail’s range are ‘edgy’ due to its small range size.

a)
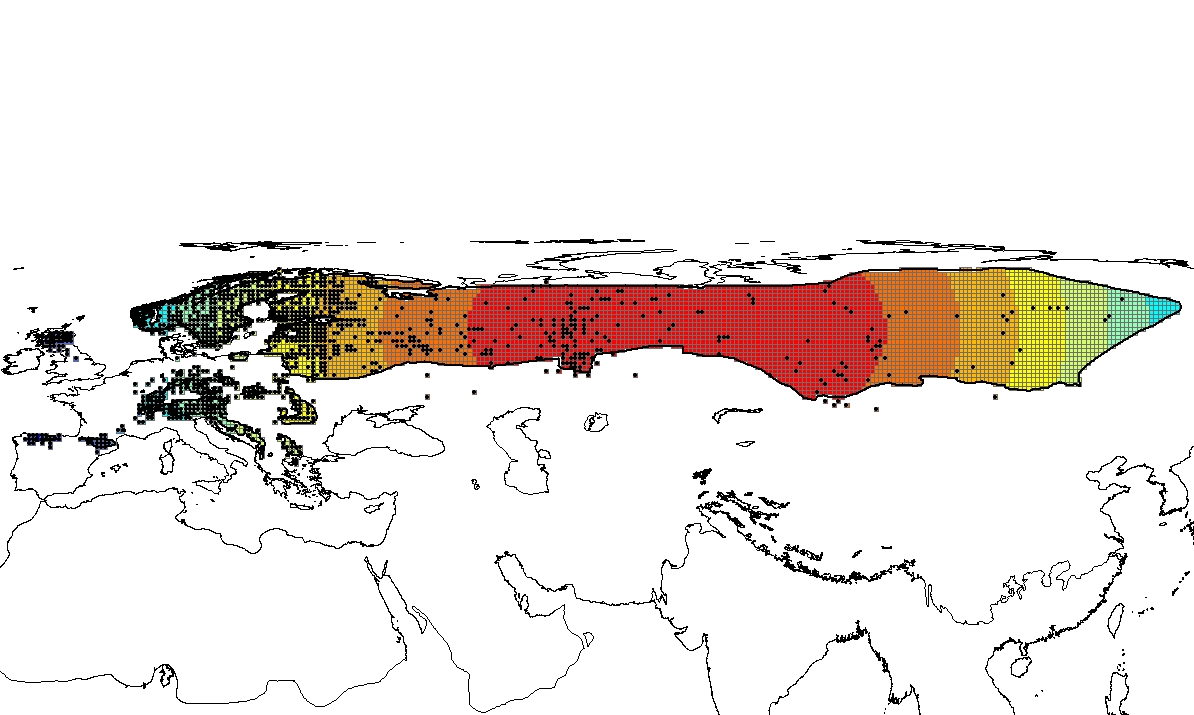


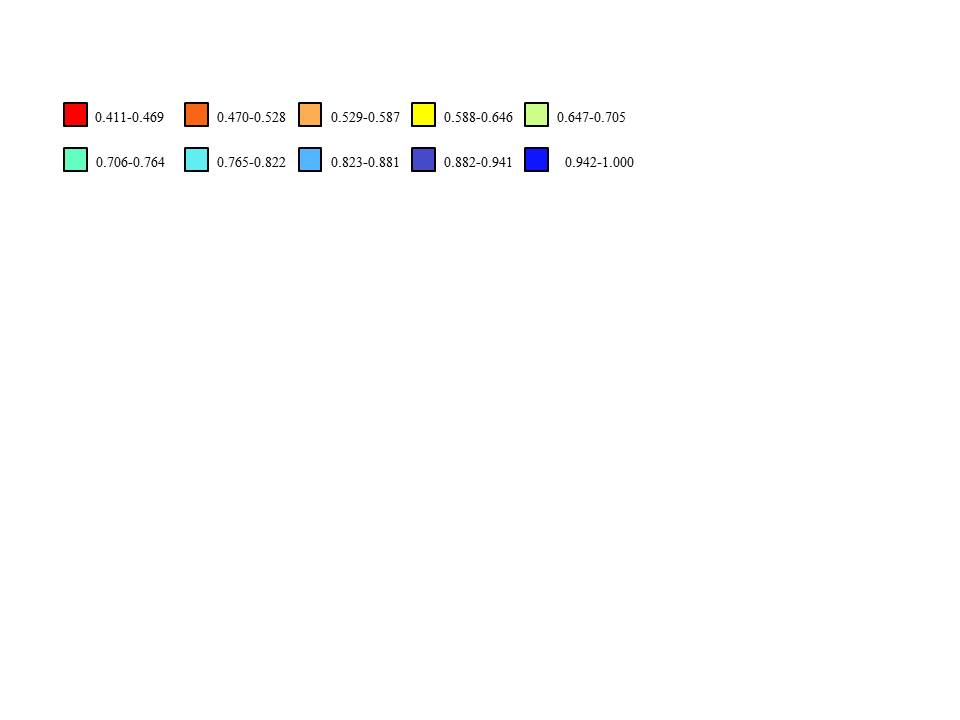


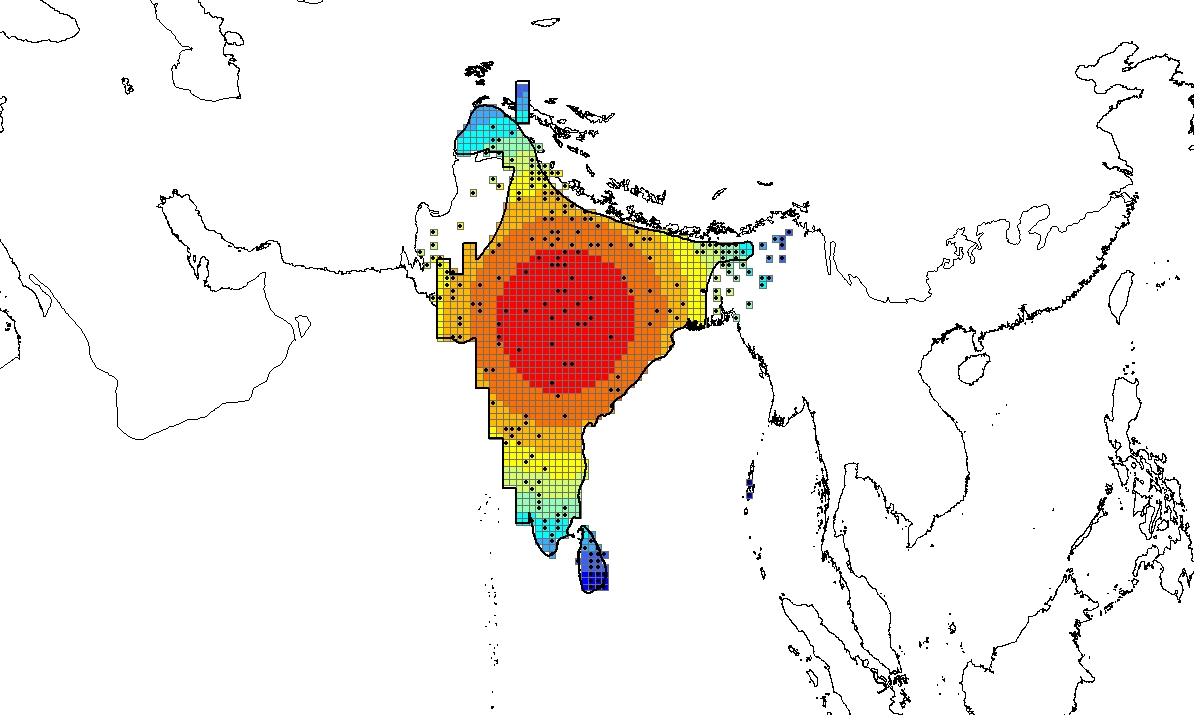


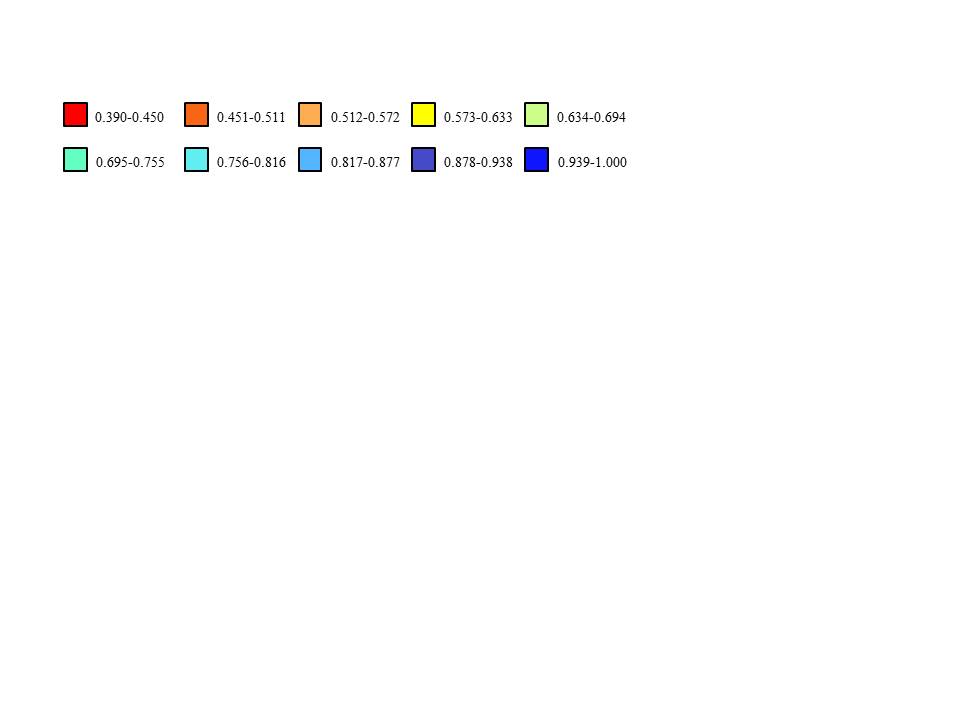


b)


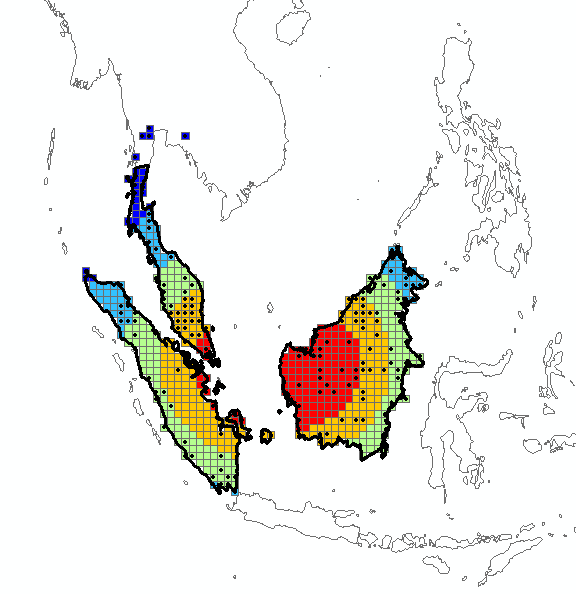


0.398-0.457

0.458-0.523

0.524-0.609

0.610-0.753

0.754-1.000

c)


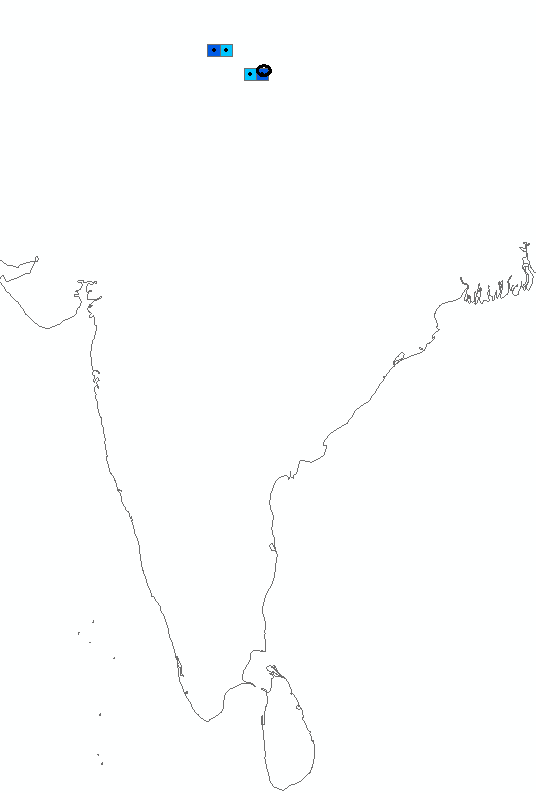


0.800 – 0.899

0.900 – 1.000

d)

Appendix S5. The land class categories for the HYDE 2.0 Land Use Data ([Klein Goldewijk, 2001](#_ENREF_1))

| HYDE Grid Code | Land class | Unconverted / Human-dominated |
| --- | --- | --- |
| 1 | Cultivated land | Human dominated |
| 2 | Pasture | Human dominated |
| 5 | Ice | Unconverted |
| 6 | Tundra | Unconverted |
| 7 | Wooded tundra | Unconverted |
| 8 | Boreal forest | Unconverted |
| 9 | Cool conifer forest | Unconverted |
| 10 | Temperate mixed forest | Unconverted |
| 11 | Temperate deciduous forest | Unconverted |
| 12 | Warm mixed forest | Unconverted |
| 13 | Grassland/steppe | Unconverted |
| 14 | Hot desert | Unconverted |
| 15 | Scrubland | Unconverted |
| 16 | Savanna | Unconverted |
| 17 | Tropical woodland | Unconverted |
| 18 | Tropical forest | Unconverted |

Appendix S6: Model validation

The figure below shows the observed and predicted number of records for each of the 14,256 cell-species combinations in our dataset, both before 1980 (red circles) and after 1980 (blue). The observed number of records is based on the 112,485 binary observations and is the number of years in which species *i* was observed in grid cell *j*. The predicted number is based on a sample from the posterior distribution of Py*_ik_,* the probability of observing species *i* on survey *k*, where k defines both the site, *i*, and time period, *t*. Py*_ik_* is the product of true occupancy, z*_ijt_* (equation 1), and the conditional probability of being recorded, p*_ik_* (equation 5). This exercise shows that there is a reasonably good correlation between observed and predicted numbers of records (Spearman’s rho = 0.732). Although the fit appears to be visually worse before 1980 (red circles) the difference is marginal (rho = 0.729 before 1980 compared with 0.731 after).
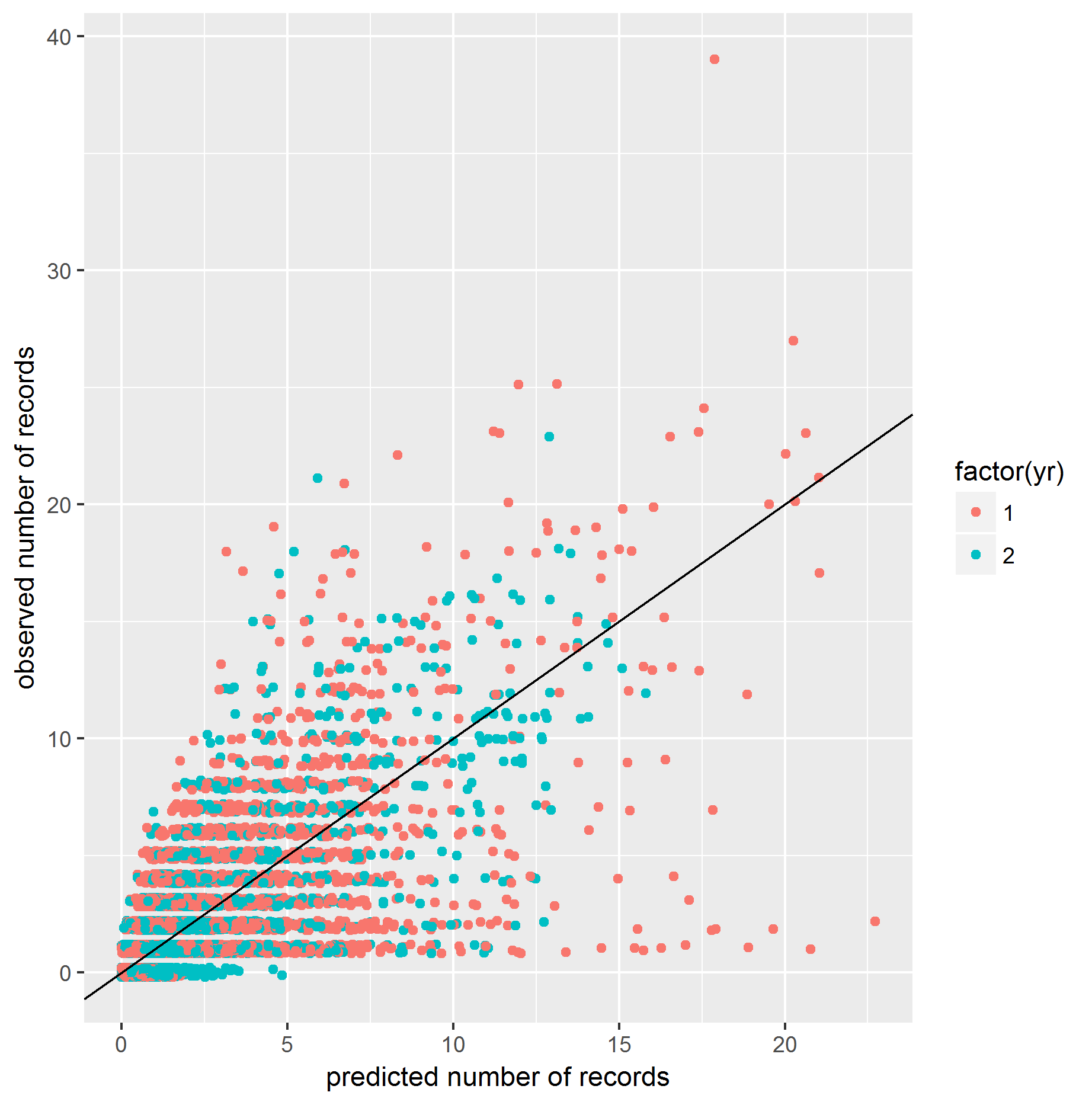


Appendix S7. The spatial distribution of a) the maximum value of *D`* for each cell. (A cell may be close to the edge of one species’ range but far from the edge of another’s.) b) The standard deviation of *D`* for each cell. The colour-scale goes from red (core) to dark blue (edge) in increments of equal interval, spanning a) 0.199 to 1.00 and b) 0.0 to 0.5.

a)


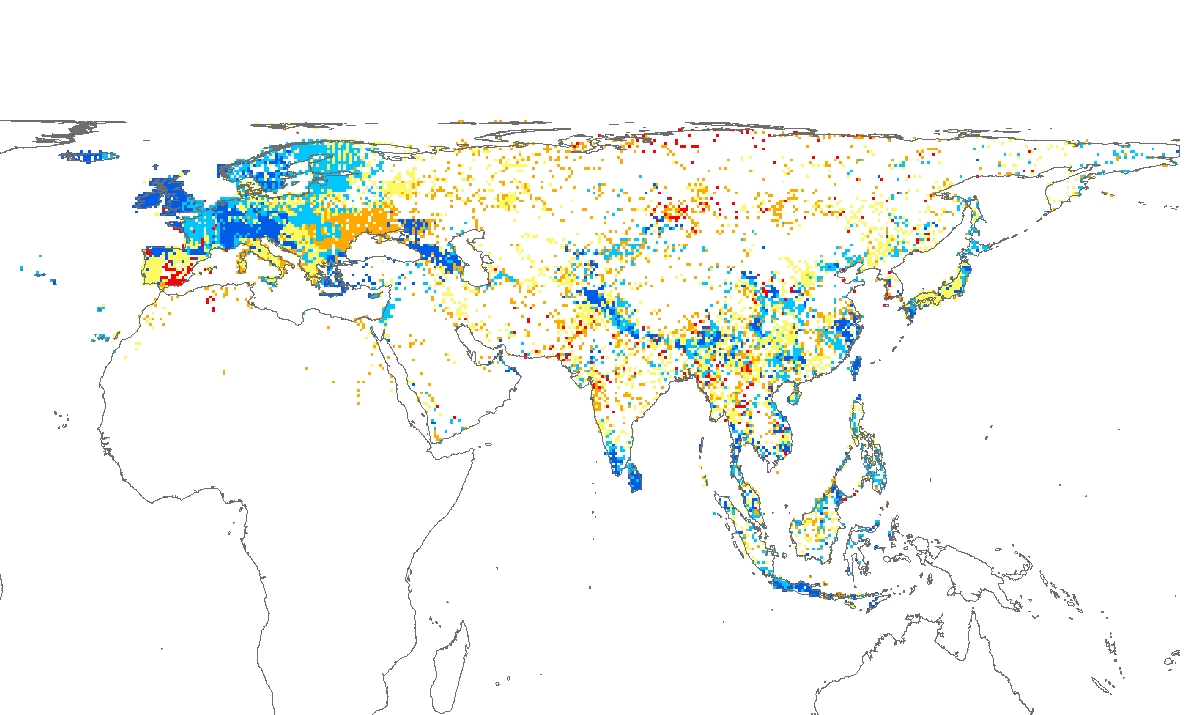


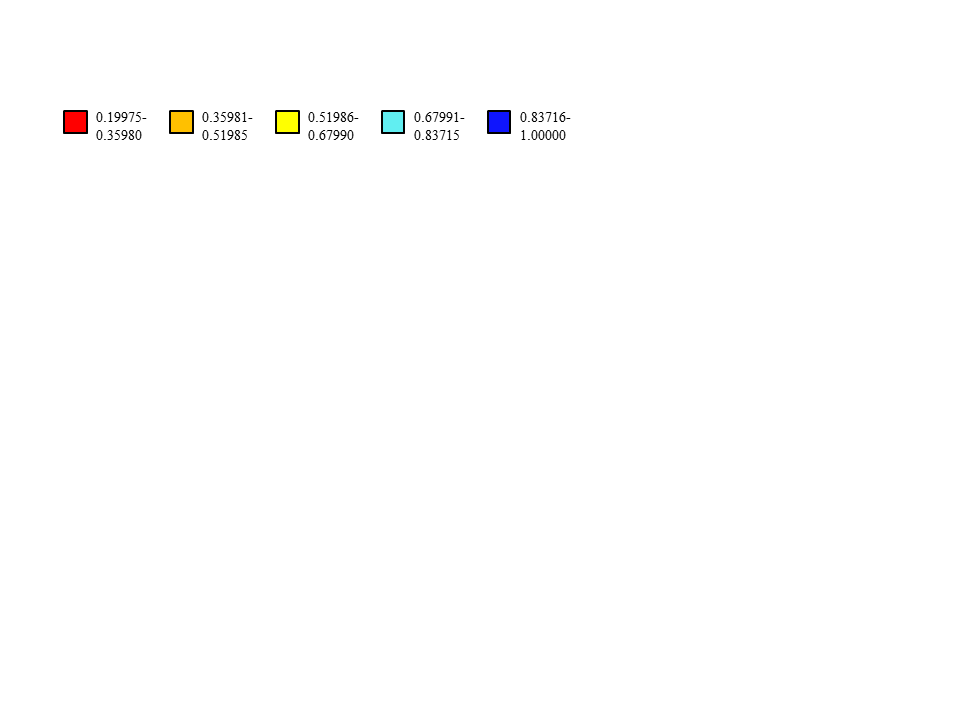


b)


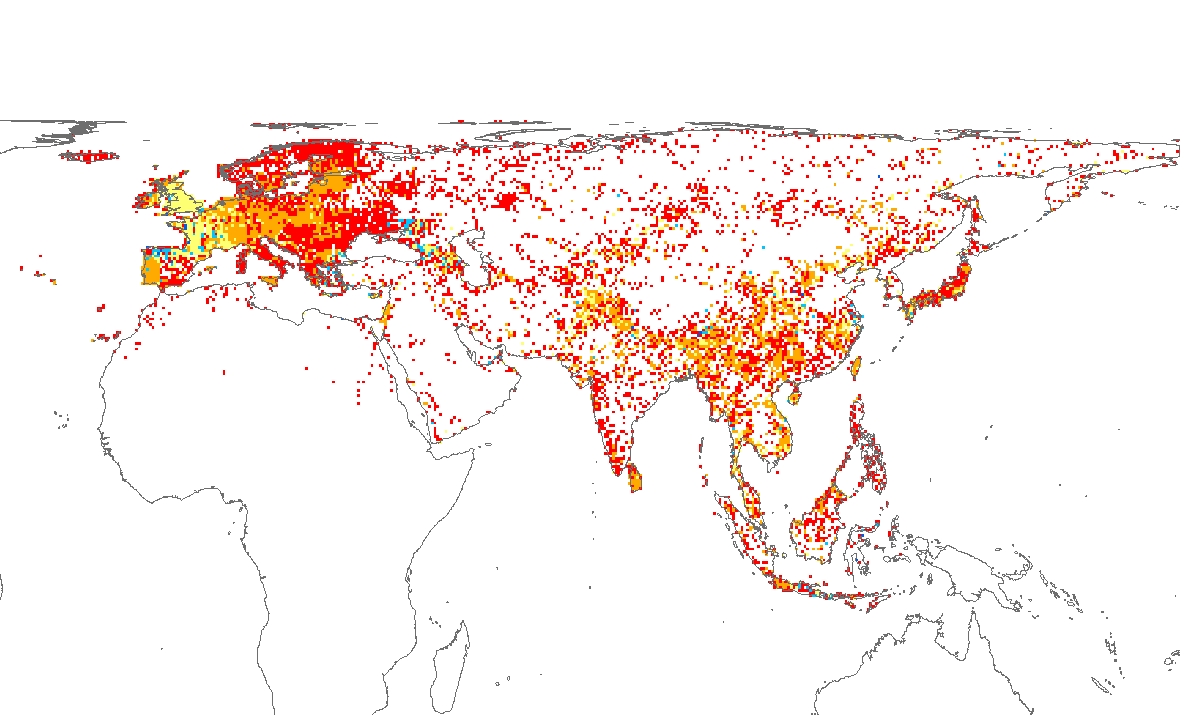


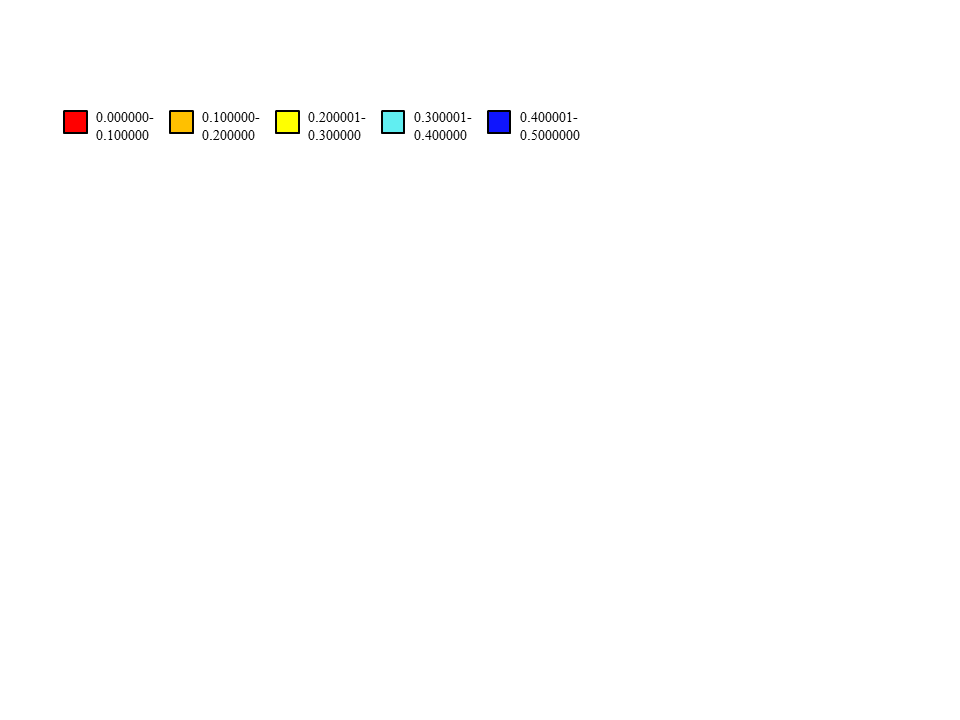


References

*Klein Goldewijk, K. (2001) Estimating global land-use change over the past 300 years: the HYDE database. Global Biogeochemical Cycles,* ***15****, 417-433.*

*Orme, C.D.L., Davies, R.G., Burgess, M., Eigenbrod, F., Pickup, N., Olson, V.A., Webster, A.J., Ding, T.-S., Rasmussen, P.C., Ridgely, R.S., Stattersfield, A.J., Bennett, P.M., Blackburn, T.M., Gaston, K.J. & Owens, I.P.F. (2005) Global hotspots of species richness are not congruent with endemism or threat. Nature,* ***436****, 1016-1019.*
